# Serelaxin activates eNOS, suppresses inflammation, attenuates developmental delay and improves cognitive functions of neonatal rats after germinal matrix hemorrhage

**DOI:** 10.1038/s41598-020-65144-4

**Published:** 2020-05-15

**Authors:** Ming M. Xu, L. Seyler, T. Bäuerle, L. S. Kalinichenko, C. P. Müller, H. B. Huttner, S. Schwab, A. Manaenko

**Affiliations:** 1 Department of Neurology, University Clinic, Friedrich-Alexander-University Erlangen-Nuremberg, Erlangen, Germany; 2 Department of Radiology, University Clinic, Friedrich-Alexander-University Erlangen-Nuremberg, Erlangen, Germany; 3 Department of Psychiatry and Psychotherapy, University Clinic, Friedrich-Alexander-University Erlangen-Nuremberg, Erlangen, Germany; 4 Department of Neurology, Xinhua hospital affiliated to Shanghai Jiaotong University School of Medicine, Shanghai, China

**Keywords:** Neuroscience, Diseases, Medical research

## Abstract

Germinal matrix hemorrhage (GMH) is a detrimental form of neonatal CNS injury. Following GMH-mediated eNOS inhibition, inflammation arises, contributing to GMH-induced brain injury. We investigated the beneficial effects of Serelaxin, a clinical tested recombinant Relaxin-2 protein, on brain injury after GMH in rats. We investigated whether effects of Serelaxin are mediated by its ability to activate the GMH-suppressed eNOS pathway resulting in attenuation of inflammatory marker overproduction. GMH was induced by intraparenchymal injection of bacterial collagenase (0.3U). Seven day old Sprague–Dawley rat pups (P7) were used (n = 63). GMH animals were divided in vehicle or serelaxin treated (3 µg once, 30 µg once, 30 µg multiple, *i.p*., starting 30 after GMH and then daily). Sham operated animals were used. We monitored the developmental profile working memory and spatial function (T-maze and open field test respectively). At day 28, all rats underwent MRI-scans for assessment of changes in cortical thickness and white matter loss. Effects of Serelaxin on eNOS pathway activation and post-GMH inflammation were evaluated. We demonstrated that Serelaxin dose-dependently attenuated GMH-induced developmental delay, protected brain and improved cognitive functions of rats after GMH. That was associated with the decreased post-GMH inflammation, mediated at least partly by amelioration of GMH-induced eNOS inhibition.

## Introduction

Germinal matrix hemorrhage (GMH) is the most common CNS injury which occurs in about 15%–20% of premature born infants weighing less than 1500 g^1,2^. The incidence of GMH dramatically increases with decreasing of the body weight of the infants and reaches up to 50% when body weight is less than 750 g^3^. Due to improved diagnostic tools and novel intensive care strategies, the mortality rate of such patients has considerably decreased over the last decades. However, the existing clinical treatments are mostly supportive and the prognoses for the future life without professional care-giving of such patients are poor. So far no causal treatment is available for treatment this severe type of neonatal brain injury.

Previously, a rat model of GMH was established^4,5^. Authors demonstrated that bleeding induced in the germinal matrix of 7 day old rat pups results in sequelae similar to those observed in human infants post GMH. Furthermore, it has been demonstrated that GMH induced overproduction of inflammatory markers shortly after impact. In the long term, inflammatory markers overproduction results in a significant loss of white matter and consequently in significant neurological dysfunctions^4–6^. In the current project we investigated whether Serelaxin is able to attenuate GMH-induced inflammation and protect the post-GMH brain of neonatal rats. Serelaxin is a clinically tested recombinant protein which in amino acid sequence and structure is identical to the naturally occurring human peptide hormone Relaxin-2. Relaxin-2 is associated with many haemodynamic and renovascular changes, which occur during the pregnancy. Previously anti-inflammatory effects of Serelaxin have been demonstrated in several preclinical and clinical studies^7^. Serelaxin, similar to Relaxin-2, mediates several molecular pathways. Most relevant for this study is the ability of Serelaxin to modulate the e-NOS pathway. eNOS is a NOS isoform mostly expressed on vascular endothelium. Activation of eNOS attenuated neurovascular changes after brain injury in adults^8^ Activation of eNOS pathway reduced brain damage by brain endothelial permeability by decreasing endothelial permeability^9^. Impairment of eNOS activity is also implicated in many cellular mechanisms of neuronal injury (for review^10^).

It has been demonstrated before that compared to relaxin 1 and relaxin 3, relaxin 2 (Serelaxin is an analog of relaxin 2) most effectively produces endothelium relaxation and that this effect is mediated via eNOS pathway^11^. Furthermore, it has been demonstrated that cardioprotective effects of serelaxin following ischemia/reperfusion injury are mediated by eNOS mediated mechanisms^12^. There are also the indications that serelaxin can attenuate cigarette smoke induced apoptosis and cell death and that this effects are mediated by ability of serelaxin to stimulate the eNOS pathway.

It has been demonstrated on the other side, that GMH results in significant inhibition of eNOS pathway, resulting decrease of eNOS phosphorylation^13^. Pharmacological activation of eNOS pathway, which increased phosphorylation of eNOS was associated with post-GMH brain protection. Authors demonstrated that GMH leads to white matter loss and decreased cortical thinkness. Activation of eNOS pathway attenuated white matter loss and increase cortical thickness after GMH. Brain protection, which was induced by eNOS phosphorylation resulted, in turn, in better neurological functions. Authors of the previous publication used the medication, which has not been proved for clinical use yet. It has not decrease the importance of their interested study, has however decrease the clinical relevance of it.

In the current study we have tested whether Serelaxin, a medication which has been already clinically tested, can decrease brain injury and improve neurological functions after GMH.

## Results

### Mortality

All pups tolerated the procedure and recovered from anesthesia. None of the sham operated animals died. However, *one out of 16 of the non-treated GMH animals and two out of eight of GMH animals threated with the low dose Serelaxin died overnight after surgery*. Hence, the mortality rate of 13% without significant differences between the different groups was observed.

### Serelaxin attenuated the GMH induced developmental delay

**Figure 1:**
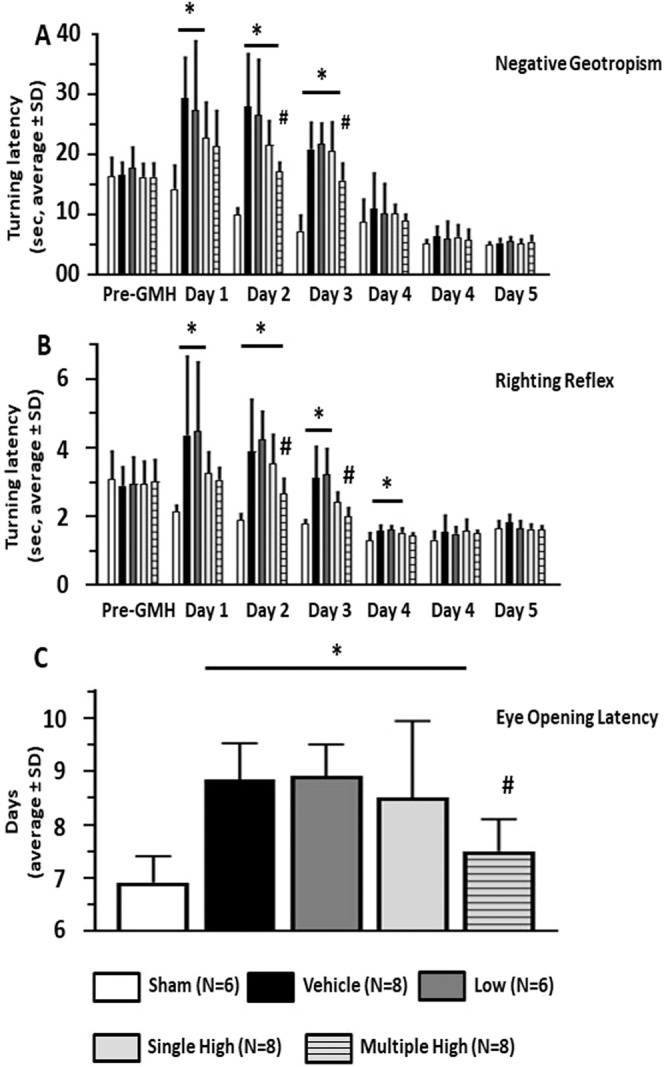
Effects of Serelaxin on developmental profile of GMH animals. Serelaxin treatment attenuated dose-dependently GMH-induced developmental delay, evaluated by Negative Geotropism (NG) (**A**) and Righting Reflex (RR) (**B**) tests as well as by monitoring of Eye-Opening Latency (**C**). Effects of GMH could be seen on days 1-3 ween evaluated by NG and on days 1-4, evaluated by RR tests *p < 0.05 compared to sham, # p < 0.05 compared to GMH + vehicle, N = 6–8/group. Data are mean ± SEM. ANOVA, Dunnett.

Compared to sham operated animals, GMH induced a significant delay in the development evaluated according to the performance of animals on negative geotropism and righting reflex tests (p < 0,05). A statistical difference between sham and GMH animals was observed till day 3 and 4, respectively, with spontaneous resolution of deficits thereafter (Fig. 1A,B, p < 0,05). While the low concentration of Serelaxin had no effects, the high concentration showed a strong tendency to decreased turning latency in both, the negative geotropism and righting reflex tests. After multiple treatment, the tendency reached significance on post GMH days 2 and 3 (p < 0,05). After single treatment the tendency did not reach statistical significance. Furthermore, the multiply treatment with high dose of Serelaxin was able to attenuate the GMH-induced latency delay of eye opening (Fig. 1C, p < 0,05).

### Serelaxin decreased GMH induced brain injury

**Figure 2:**
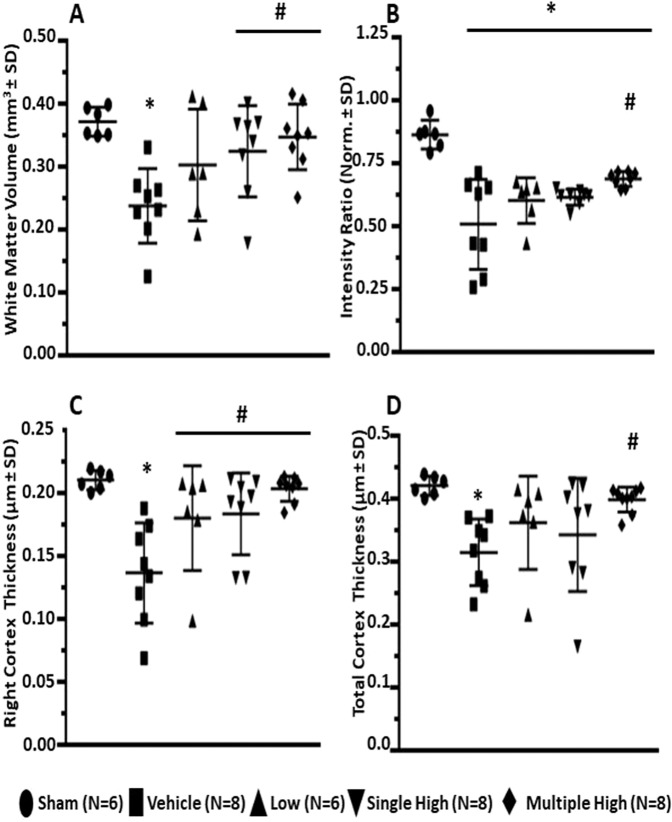
Effects of Serelaxin on post-GMH brain injury. Serelaxin treatment diminished dependently GMH-induced white matter loss (**A,B**) and increased cortical thickness of treated animals (**C,D**), *evaluated by MRI study 28 days after GMH*. * p < 0.05 compared to sham, # p < 0.05 compared to GMH + vehicle, N = 6–8/group. Data are mean ± SEM. ANOVA, Dunnett.

A significant loss of white matter was observed in non-treated GMH animals compared to sham operated animals (Fig. 2A, p < 0.05) at day 28. GMH was associated with a decrease of (white matter)/cortex signal intensity ratio (Fig. 2B) and a reduction of cortical thickness (Fig. 2C,D, p < 0,05). Treatment with high concentration of Serelaxin attenuated GMH-induced white matter loss and changes of (white matter)/cortex signal intensity, whereby multiple treatments with Serelaxin proved itself as most effective therapeutic strategy (Fig. 2A,B, p < 0,05). Similarly, Serelaxin also increased the cortical thickness of GMH animals (Fig. 2C,D, p < 0,05).

### Serelaxin improved working memory and spatial functions of GMH animals

**Figure 3:**
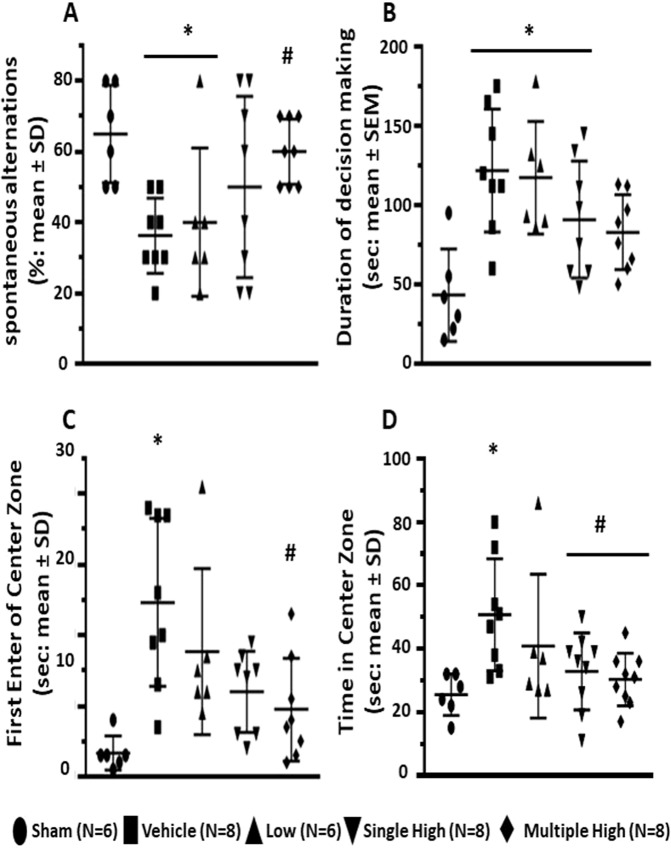
Effects of Serelaxin on spatial function of GMH animals. *Open field test, conducted on 27*^*th*^ *day after GMH induction demonstrated* that Serelaxin treatment reversed GMH-induced increase of both central locomotion and time (**A**), spent by animals in the center zone (**B**). Furthermore, the treatment improved working memory (T-Maze test, 26^th^ day after GMH), evaluated by monitoring of spontaneous alteration (**C**) and time, needed for doing a decision (**D**). * p < 0.05 compared to sham, # p < 0.05 compared to GMH + vehicle, N = 6–8/group. Data are mean ± SEM. ANOVA, Dunnett.

We demonstrated that GMH negatively affected working memory of the animals, which resulted in the decreased level of spontaneous alternations in the T-Maze test (Fig. 3A). The working memory was evaluated at post GMH day 26. Furthermore, GMH animals compared to the sham animals needed more time for a decision making of which arm of the maze they enter (Fig. 3B). Serelaxin dose dependently reversed deleterious effects of GMH. While the low concentration had no effects, the high concentration increased the number of spontaneous alterations. There was only an insignificant difference in decision time between sham operated and animals treated multiply with high doses of Serelaxin (Fig. 3A,B, p < 0,05). Similarly, GMH compared to sham-operated animals needed more time to make a decision to enter center zone of open field apparatus (post GMH day 27) (Fig. 3D, p < 0,05). Although they enter the zone later, GMH animals spent more time in the center then sham operated animals (Fig. 3C, p < 0,05). Serelaxin reversed these parameters. Animals treated with high concentration of Serelaxin entered the center zone quicker, but spend less time in the zone.

### Serelaxin reversed effects of GMH on grooming and rearing behavior

**Figure 4:**
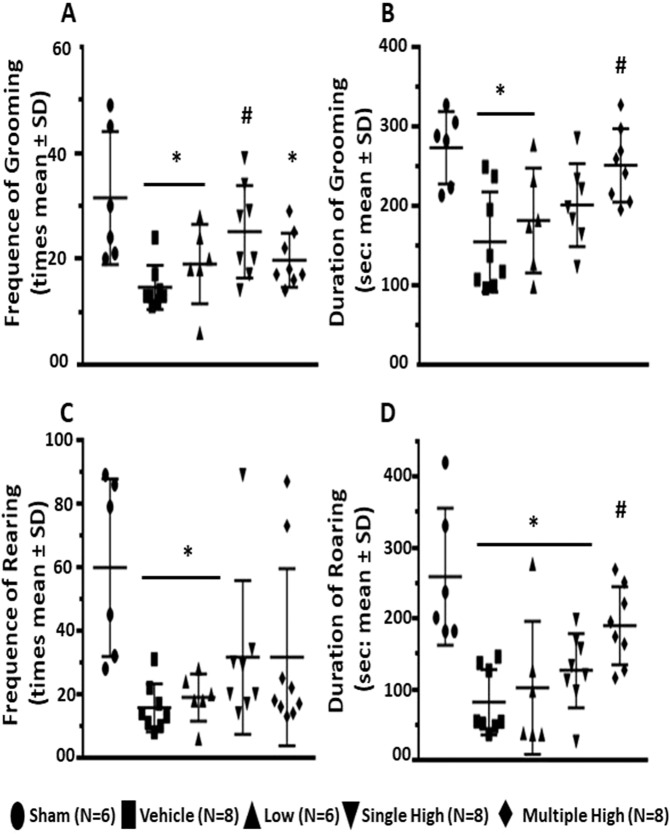
Effects of Serelaxin on Grooming and Rearing behavior of GMH animals. As evaluated at day 27, Serelaxin treatment was associated with increased incidence and duration of both Grooming (**A,B** respectively) and Rearing behavior (**C,D** respectively). *p < 0.05 compared to sham, # p < 0.05 compared to GMH + vehicle, N = 6–8/group. Data are mean ± SEM. ANOVA, Dunnett.

On post GMH day 27, GMH resulted in a significant decrease of episodes and time of grooming and rearing behavior of GMH animals compared to sham operated animals (Fig. 4A–D, p < 0.05). Treatment of animals with low concentration of Serelaxin had no effect. On the contrary, treatment of animals with high concentration of Serelaxin increased these parameters.

### Serelaxin reversed effect of GMH on the phosphorylation of NOS

**Figure 5:**
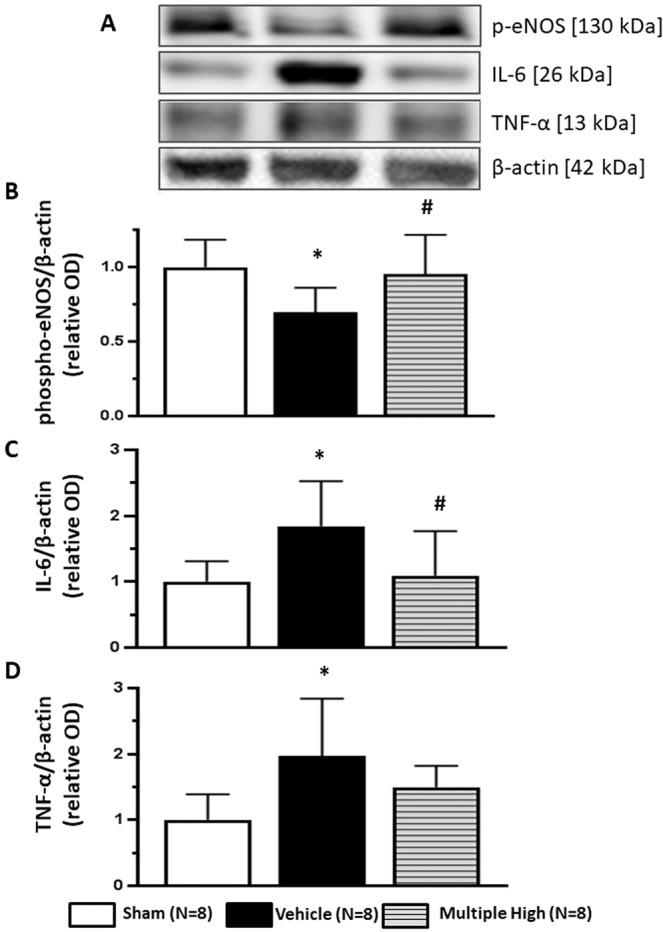
Effects of Serelaxin on post-GMH brain inflammation. (**A**) Representative western blot results. 72 hours after GMH significant decrease of e-NOS phosphorylation in brain of none treated compared to sham operated animals was observed (**B**). Consequently, overproduction of inflammatory factors IL-6 and TNFα in the brain was induced (**C**,**D** respectively). Serelaxin reversed GMH effect on e-NOS phosphorylation, decreasing brain inflammation after GMH. *p < 0.05 compared to sham, #p < 0.05 compared to GMH + vehicle, N = 8/group. Data are mean ± SEM. ANOVA, Dunnett.

GMH significantly decreased phosphorylation of the NO-Synthase endothelial isoform, eNOS, after 72 hours (Fig. 5A, p = 0.015). That was accompanied with an overproduction of the inflammatory markers, IL-6 and TNF-α (Fig. 5B,C, p = 0.022 and p = 0.007 respectively), also evaluated 72 hours after GMH. Multiple administration of Serelaxin attenuated the GMH-induced overproduction of IL-6 and TNF-α, evaluated 72 hours after GMH. However, only by Il-6 the difference compared to the none treated animals reached significance (p = 0.043).

## Discussion

GMH, is a most detrimental event, which affect more than 40% of premature born infants with extreme low body weight^1^.

Mounting evidences demonstrated that inflammation is one of the factors which undoubtfully contributes to the development of post GMH brain injury^13–16^. In this project we evaluate whether a therapy with the clinically tested medication Serelaxin can attenuate post-GMH inflammation, protecting the brain, attenuating developmental delay and improving cognitive function of rats after GMH.

We induced GMH on 7 days old rat pups. This age is roughly equivalent to the 30–32 week preterm human neonate^17^. We used a previously described a rat “collagenase” model of GMH^4,5^. A significant advantage of the model is the similarity in sequels between rat pups after collagenase injection and human infant suffering GMH. In human infants hemorrhage generally begins with the rupture of blood vessels within the germinal-matrix^18^. Filling of ventricular system with blood, upon breaking of ependyma, follows. Similarly, in rat pups an injection of collagenase induces spontaneous and progressive escalation of blood-vessel rupture, focal bleeding and re-bleeding^5,19^. Authors of previous publications demonstrated that initial, collagenase-induced bleeding results in short term neurological dysfunctions^4,5^. Furthermore, GMH induced a strong inflammatory response, which in the long term was associated with white matter loss and in a decrease of cortical thickness^5^. That, in turn, was associated with development of cognitive dysfunctions and working memory impairments^13,20^.

It is important to mention, that hypothermia is able to significantly attenuate the development of neonatal brain injury both in humans and in pre-clinical models^21,22^. In fact, hypothermia has become standard clinical treatment after perinatal asphyxia^23^. Therefore, the monitoring of body temperature is important in this model and the body temperature was continually controlled throughout the whole surgery.

In the first part of the project we demonstrated that GMH delayed the development of rat pups after GMH, resulting in increased time on the negative geotropism test and righting reflex evaluation. Furthermore, the GMH increased the eye open latency. This is in agreement with previously obtained results, which also used similar strategy of post GMH developmental profile evaluation^1,24,25^.

The goal of this study was the investigation whether Serelaxin would be able to protect the neonatal brain from GMH-induced injury and attenuate post-GMH developmental delay and neurological dysfunctions. Serelaxin is a recombinant protein which is in its amino acid sequence and structure identical to the naturally occurring human peptide hormone Relaxin-2, a hormone which is associated with many haemodynamic and renovascular changes occuring during pregnancy^26,27^. Serelaxin (known as Reasanz) is marketed for the treatment of acute heart failure in Russia. It has been demonstrated that compared to placebo, serelaxin reduced worsening heart failure (WHF) and cardiovascular mortality^28^. Serelaxin has undergone clinical trials in patients with acute heart failure^29,30^. Although serelaxin failed in globale Phase-III-trail RELAX-AHF-2, the safety and tolerability profile of the drug has been well established^31–33^.

It has been demonstrated previously that the treatment with human relaxin 2 is able to reduce hypertrophy and apoptosis in neonatal rat cardiomyocytes, which were challenged with H_2_O_2_^34^. Also, there is an indication that relaxin 2 can promote cardiac regeneration in neonates by modulating growth and promoting maturation of neonatal cardiomyocytes^35^. Presents of relaxin receptors during embryo development, and beyond have been demonstrated before^36,37^. These findings let us to the hypothesis that treatment with a Relaxin 2 analog, Serelaxin could have a beneficial effect after neonatal brain injury.

We tested different concentrations of Serelaxin and were able to demonstrate that a high concentration of the drug significantly improved performance of GMH animals on negative geotropism test and righting reflex and decreased eye opening latency of GMH pups. This is also in agreement with previous publications, which demonstrated that anti-inflammatory treatments may result in an attenuation of GMH-induced developmental delay^20,38^. The half life time of Serelaxin in blood is 6-8 hours in humans and 4–6 hours in rats^12,39^. Authors of previous publications, which investigated the effects Serelaxin on brain injury, either used a micro pump for continuous delivery of Serelaxin over a long period of time or evaluated the effects of the drug in short (4 hours) term studies^40–43^. In our preliminary experiments we figured out that P7 rat pups tolerate implantation of a mini pump rather badly. Therefore, we tested the effects of multiple drug administration, first 30 minutes and then one time daily over a period of three days. Using this strategy we demonstrated that multiple administration of a high dose of Serelaxin was more effective and induced longer lasting effects compared to single administration of a high dose of Serelaxin.

It has been demonstrated previously, that pro-inflammatory effects of GMH are mediated, at least partly, by its ability to inhibit the eNOS pathway (eNOS is a NOS isoform mostly expressed on vascular endothelium) and that the pharmacological activation of the pathway is accompanied with a decrease in the production of the pro-inflammatory mediators, TNF-α and IL-6^13,44^.

In the current project we tested potentially protective effects of Serelaxin on GMH-induced brain injury and neurological dysfunctions. In preclinical and clinical studies numerous actions of Serelaxin have been demonstrated^41^. For the current project, the anti-inflammatory effects of Serelaxin are most relevant. Previously, it has been demonstrated that both Relaxin-2 and Relaxin-3 can effectively decease infarct size after temporal middle cerebral artery occlusion (tMCAO)^42,43^. Authors also demonstrated that the stroke-induced inhibition of endothelial nitric oxide synthesis completely abolished protective effects of Relaxin^20^. Others also demonstrated that effects of both Relaxin-2 and Serelaxin are meditated by their ability to activate eNOS, but not another isoform of the NOS signaling pathway^11,45^. The activation of eNOS is neuroprotective after stroke and, as mentioned above, after GMH^46^. Other studies on GMH used for eNOS activation a clinically not tested drug. That does not decrease the scientific value of the obtained data, however, the translational significance is limited^1^. On the contrary, Serelaxin has been clinically approved and testing of clinically approved drugs may always bear a higher translation value.

In the second experiment, we tested the effect of Serelaxin treatment on GMH-induced brain damage and consequently on impairment of working memory and cognitive functions. The severity of the brain damage was evaluated in an MRI study. It has been demonstrated that the development of GMH leads to inflammation damaging the periventricular white matter, apoptosis and arresting maturation of oligodendrocyte precursor cells, and hypomyelination^47^. Negative effects of inflammation, resulting in the white matter loss have been observed in both preclinical and clinical studies^48–50^. Infants suffering GMH are prone to disrupted white matter maturation and impaired cognitive development^51^. In concord with others we demonstrated that GMH resulted in a significant loss of white matter and decreased cortical thickness^5,52^. Serelaxin treatment, similarly to other anti-inflammatory treatments, attenuated both, the GMH-induced white matter loss and the decrease in cortical thickness^25,38^.

The effects of treatment on working memory were evaluated by T-Maze test. In concord with others we showed that collagenase induced GMH led to worsening of working memory resulting in decreased number of spontaneous alterations^4,53^. Serelaxin dose dependently attenuated the GMH-induced disruption of working memory. Furthermore, GMH attenuated the ability of animals to make a decision, resulting in increased time between start of test and entering of one of the maze arms.

Cognitive functions of animals were evaluated by open field test. Animals after GMH are hyperactive and cover longer distances in the open field^4^. Correspondently, GMH animals in the current project spent more time in the center zone. Interesting enough, GMH animals needed more time for entering the center zone for the first time. That supported our T-Maze test results, demonstrating that GMH compared to naïve animals were not willing to explore the surroundings and needed more time to enter one of the T-Maze arms. Hyperactivity of GMH animals, observed before, corresponded with other results of the open field test in the current project. Increased horizontal activity of these animals limits their vertical activity, resulting in decreased frequency and time of rearing behavior. Furthermore, horizontal hyperactivity of GMH animals decreased their alacrity to groom themselves, resulting in smaller frequency and less time spent grooming. In agreement with MRI study results, Serelaxin-induced protection resulted in improved working memory and smaller cognitive dysfunctions after GMH.

In the last part of the project we investigated whether the observed protective effects of Serelaxin are indeed mediated by its anti-inflammatory capacity. Since the importance of the eNOS pathway in the development of post-GMH brain injury has been demonstrated before, we focused on eNOS pathway^25^. Previously it has been demonstrated that GMH did not change the production of eNOS^25^. However, similar to other injuries, GMH decreased phosphorylation of eNOS^25^. We tested the effects of the multiple administration of Serelaxin, which proved itself as most effective strategy in previous experiments, on eNOS phosphorylation. Well in agreement with other reports, Serelaxin attenuated stress-induced changes in eNOS phosphorylation^12,43^. The GMH-induced decrease of eNOS phosphorylation was accompanied with overproduction of TNF-α and IL-6. This overproduction, in turn, was accompanied with brain injury and white tissue loss^13^. Therefore it was not surprising that in the current project that neuroprotective effects of Serelaxin were associated with its ability to reverse the GMH-induced inhibition of eNOS phosphorylation, which, in turn, decreased overproduction of pro-inflammatory cytokines in neonatal rat brains after GMH.

It is worth to mention, that GMH is also able to change the production of other isoform of NOS^18^. Moreover, Serelaxin can activate both NOS pathways and prostanoids pathway^45,54^. Our publication we demonstrated to the first time that Serelaxin protects neonatal brain after GMH. Further investigations of molecular pathways, underlying protective effects of Serelaxin, which were observed in our study are urgently needed.

## Conclusion

We demonstrated for the first time that clinically proved recombinant human Relaxin 2, Serelaxin, is able to protect neonatal brain after GMH. This protection is provided by an attenuation of GMH-induced white matter loss, cortical thickness decrease, and improvement of cognitive functions. The beneficial effects of Serelaxin were accompanied by an activation of the eNOS pathway and a decrease of brain inflammation after GMH.

## Materials and Methods

### Animal groups and general operative procedures

The study was conducted in accordance with the ARRIVE (Animal Research: Report of *In Vivo* Experiments) guidelines^55^ and conducted in accordance with the Federation of European Laboratory Animal Science Associations (FELASA) guidelines. All experiment protocols have been approved by institutional review board of pre clinical experimental center of University Clinic (Universitätsklinikum, Friedrich-Alexander-Universität Erlangen-Nürnberg) and carried out according with §15 of european animal protection law and in agreement with recommendation of Animal Welfare Commission of Government Unterfranken (RL 3010/63/EU, Animal protocol number 55.2-2532-2-550).

Pregnant Sprague–Dawley rats were obtained from Charles River Laboratories (Sulzfeld, Germany) and housed in a specialized animal care facility (Franz-Penzoldt-Zentrum, University Erlangen-Nuremberg, Erlangen, Germany) in a room with constant temperature (25 °C), humidity control and a 12/12 h light/dark cycle with free access to food and water.

Sixty-three P7 rat pups of both genders were randomly assigned to the following groups: sham-operated (n = 14), GMH-vehicle treated (n = 17) and GMH-Serelaxin treated (N = 32) animals (for study design see Supplemental Fig. 1). Animals treated with Serelaxin were further randomly divided in threated with low, single or multiple dose of Serelaxin. Rats were subjected to the operative procedure, using aseptic technique as described before^5,56^. Anesthesia was inducted with 3.0% isoflurane (in mixed air and oxygen). Afterwards, the rat pups were placed prone onto a stereotaxic frame and concentration of isoflurane was increased till 1.5%. The body temperature of 37 °C was maintained using an electronic thermostat-controlled warming blanket. For GMH induction, betadine first sterilized the surgical scalp area, which was then incised in the longitudinal plane to expose the skull and reveal the bregma. The stereotactic coordinates were measured from bregma: 1.8 mm (rostral), 1.5 mm (lateral). A burr hole (1 mm) was drilled and a 27 gauge needle was slowly inserted at a rate of 1 mm/min at the depth of 2.8 mm from the dura. Using a microinfusion pump (Harvard Apparatus, Holliston, MA) 0.3 units in 0,5 µl of clostridial collagenase VII-S (Sigma, St Louis, MO) was infused through the Hamilton syringe. The needle remained in place for an additional 10 minutes after injection to prevent “back-leakage”. After needle removal, the burr hole was sealed with bone wax, the incision suture closed, and the animals were allowed to recover on a 37 °C heated blanket. Upon recovering from anesthesia, the animals were returned to their dams. Sham surgery consisted of needle insertion alone without collagenase infusion.

One vehicle treated and two Serelaxin low dose treated animals died overnight after surgery. Vehicle treated animals was replaced and used for western blot studies.

### Drug administration and animal groups

The stock solution was kindly provided by Novartis Pharma GmbH. Working solutions were prepared on the day of the experiment. GMH animals were randomly divided in groups treated with sterile saline (200 µl, vehicle group), treated once 30 min after GMH with a low dose (3 µg/kg of body weight, total volume 200 µL), or with a high dose (30 µg/kg of body weight, total volume 200 µL), or with multiple administration of Serelaxin (30 µg/kg of body weight; total volume 200 µL 30 minutes after GMH and then daily for 2 days). For treatment schedule see: Suppl. Information. Sham operated animals received intraperitoneal injection of sterile saline (200 µl, 30 minutes after surgery).

### Monitoring of developmental profiles following GMH

The developmental profiles were monitored for 10 days. The monitoring was conducted by a researcher blind to the treatment in a random and biased setup. Negative geotropism and righting reflex tests were conducted pre-operative and 5 days after surgery. For negative geotropism, rat pups were placed on a slope (20° angle) with head pointing downwards. The time needed for the animals to turn 180° was recorded. The maximum duration of the test was 60 s per trial. 3 trails per animal per days were conducted. The average time was calculated^5,13,56^.

For righting reflex, pups were placed on their back. The time required for the rat pups to completely rollover onto all four limbs was measured. The maximum duration of the test was 60 s per trial. Three trails per animal per days were conducted. The average time was calculated^5,13,56^. For evaluation of eye opening latency animals were observed for 10 days, twice a day, every 12 hours.

### Monitoring of mental and cognitive functions following GMH

#### T-Maze

The T-Maze assesses short-term (working) memory ability. The test was conducted 26 days after GMH. Animals were placed into the start arm (40 cm × 10 cm) of the T-maze and allowed to explore it until either the left or right arm was chosen. 10 trials per animals were conducted. The rate of spontaneous alternation (0% = none and 100% = complete; alternations/trial) was tabulated. The time from the beginning of the test till an animal completely entered the arm was counted as time needed for decision making^57–59^.

#### Open field

Open field test was conducted 27 days after GMH. A black square box (50 cm × 50 cm each) was used. Transmission of olfactory cues was prevented by thorough cleaning with ethanol (70%) after each session. Rats (4 weeks old) were placed in the center of the arena and allowed to explore it freely for 20 min. For analysis, each arena was virtually split “corners,” and “center.” Activity of animals was recorded. Parameters such as, 1) the time needed to enter the “center” zone for the first time, 2) the time spent in the “center” zone, 3) the frequency and duration of rearing behavior, and 4) the frequency and duration of grooming behavior were measured manually^4,24,60^. The moment when both forepaws crossed the imaginary line was counted as enter the “center zone”. The moment when all paws crossed the line was counted as an exit off the “center zone”.

### Magnetic resonance imaging

MR-Imaging was used to investigate effect of GMH and treatments on white matter (Corpus Callosum) volume, [white matter/cortex] intensity ratio and thickness of the cortex 28 days after GMH.

A small-animal MRI system (ClinScan 70/30, Bruker BioSpin MRI GmbH, Ettlingen, Germany) equipped with a dedicated rat brain coil was used for image acquisition. Animals were anesthetized with isoflurane (1.5%) prior to the procedure. During the MRI scan an animal monitoring system was used for surveillance of heart and lung functions. The body core temperature was kept constant during the procedure at 37 °C using a heated pad. The volume and the signal intensity of the structures given above were calculated by measuring the respective regions of interest using Chimaera’s segmentation tool (Chimaera GmbH). The following T2 Turbo Spin Echo sequence was used: Time of acquisition 5:51 min, voxel size 0,098 × 0,098 × 0,5 mm, TE:41 ms, TR: 6160 ms, slice thickness 0.5 mm, 35 slices. For representative MRI scan please see Supplemental Fig. 2.

### Western blot

For evaluation of Serelaxin effects on post-GMH inflammation a separate cohort of animals (N = 24) was operated. Animals were randomly divided into sham operated, GMH vehicle treated, as well as GMH animals multiply treated with Serelaxin (30 µg/kg of body weight, treatment starting 30 minutes after GMH and then twice, every 24 hours). 72 hours after GMH animals were finally anesthetized with an overdose of ketamine and intracardially perfused with 100 ml of cold PBS. Hemispheres were isolated and stored at -80 °C until analysis. The ipsilateral hemispheres were used for the further analysis and latter proceeded as described before^13,56^. The whole-cell lysates were obtained by gently homogenizing in RIPA lysis buffer (Santa Cruz Biotechnology, Inc., sc-24948) and centrifuging (14,000 g at 4 °C for 30 min). The supernatant was collected and the protein concentration was determined using a detergent compatible assay (Bio-Rad, Dc protein assay). Equal amounts of protein (30 µg) were loaded and subjected to electrophoresis on an SDS-PAGE gel. After being electrophoresed proteins were transferred to a nitrocellulose membrane. The quality of the protein transfer was proved with Ponceau S solution (Sigma Aldrich) and areas of interest were separated from every membrane according the molecular weight marker (Sigma Aldrich) and molecular weight of target protein, provided by vendors. The membrane strips were blocked and incubated with the primary antibody overnight at 4 °C. The following primary antibodies were used:

Rabbit polyclonal to Phospho-eNOS (Ser1177) 1/500 (ThermoFisher, PA5-17917).

Rabbit polyclonal to TNF-α 1/100 (Abcam, ab6671).

Rabbit polyclonal to IL-6 (Abcam ab6671).

After cutting of the area of interest the same membrane was probed with an antibody against β-actin (Santa Cruz, 1:1000) for an internal control. Incubation with both primer and with secondary antibodies (goat anti-rabbit Jackson immuno laboratories 111-035-003) was done overnight at 4 °C. Immunoblots were then probed with an ECL Plus chemiluminescence reagent kit (Amersham Biosciences, Arlington Heights, IL) and analyzed by FusionFX (Peqlab, Erlangen, Germany) and quantified using the Bio1D Analyzer software (Vilber Lourmat, Eberhardzell, Germany).

For results of western blot study please see Supplemental Fig. 3.

### Statistical analysis

All the data were presented as mean ± SEM. Significance was considered at P < 0.05. Behavior data were statistically analyzed using one-way analysis of variance (ANOVA), followed by the Dunnett’s multiple comparisons test. Statistical analyses were performed using GraphPad Prism 7.00.

### Compliance with Ethics Requirements

All institutional and national guidelines for the care and use of laboratory animals were followed.

## Supplementary information

Supplementary information.

## References

[1] Hefti MM (2016). A Century of Germinal Matrix Intraventricular Hemorrhage in Autopsied Premature Infants: A Historical Account. Pediatr. Dev. Pathol..

[2] Lin PY, Hagan K, Fenoglio A, Grant PE, Franceschini MA (2016). Reduced cerebral blood flow and oxygen metabolism in extremely preterm neonates with low-grade germinal matrix- intraventricular hemorrhage. Sci. Rep..

[3] Itsiakos G (2016). Intraventricular Hemorrhage and Platelet Indices in Extremely Premature Neonates. J. Pediatr. Hematol. Oncol..

[4] Lekic T, Manaenko A, Rolland W, Tang J, Zhang JH (2011). A novel preclinical model of germinal matrix hemorrhage using neonatal rats. Acta Neurochir. Suppl..

[5] Lekic T (2012). Rodent neonatal germinal matrix hemorrhage mimics the human brain injury, neurological consequences, and post-hemorrhagic hydrocephalus. Exp. Neurol..

[6] Strahle J (2012). Mechanisms of hydrocephalus after neonatal and adult intraventricular hemorrhage. Transl. Stroke Res..

[7] Sanchez-Mas J (2017). Early Anti-inflammatory and Pro-angiogenic Myocardial Effects of Intravenous Serelaxin Infusion for 72 H in an Experimental Rat Model of Acute Myocardial Infarction. J. Cardiovasc. Transl. Res..

[8] Hafez, S., Khan, M. B., Awad, M. E., Wagner, J. D. & Hess, D. C. Short-Term Acute Exercise Preconditioning Reduces Neurovascular Injury After Stroke Through Induced eNOS Activation. *Transl Stroke Res*, 10.1007/s12975-019-00767-y (2019). 10.1007/s12975-019-00767-y 31858409

[9] Zhang S (2019). Myricetin ameliorated ischemia/reperfusion-induced brain endothelial permeability by improvement of eNOS uncoupling and activation eNOS/NO. J. Pharmacol. Sci..

[10] Garry PS, Ezra M, Rowland MJ, Westbrook J, Pattinson KT (2015). The role of the nitric oxide pathway in brain injury and its treatment–from bench to bedside. Exp. Neurol..

[11] Lian X (2018). RXFP1 Receptor Activation by Relaxin-2 Induces Vascular Relaxation in Mice via a Galphai2-Protein/PI3Kss/gamma/Nitric Oxide-Coupled Pathway. Front. Physiol..

[12] Valle Raleigh J (2017). Reperfusion therapy with recombinant human relaxin-2 (Serelaxin) attenuates myocardial infarct size and NLRP3 inflammasome following ischemia/reperfusion injury via eNOS-dependent mechanism. Cardiovasc. Res..

[13] Zhang Y (2018). Bliverdin reductase-A improves neurological function in a germinal matrix hemorrhage rat model. Neurobiol. Dis..

[14] Zhang, Y. *et al*. Chemerin suppresses neuroinflammation and improves neurological recovery via CaMKK2/AMPK/Nrf2 pathway after germinal matrix hemorrhage in neonatal rats. *Brain, behavior, and immunity*, 10.1016/j.bbi.2018.02.015 (2018). 10.1016/j.bbi.2018.02.015 PMC5953818 29499303

[15] Park SH (2006). Neonatal brain damage following prolonged latency after preterm premature rupture of membranes. J. Korean Med. Sci..

[16] Salafia CM (1995). Maternal, placental, and neonatal associations with early germinal matrix/intraventricular hemorrhage in infants born before 32 weeks’ gestation. Am. J. Perinatol..

[17] Tucker AM, Aquilina K, Chakkarapani E, Hobbs CE, Thoresen M (2009). Development of amplitude-integrated electroencephalography and interburst interval in the rat. Pediatr. Res..

[18] Conner ES, Lorenzo AV, Welch K, Dorval B (1983). The role of intracranial hypotension in neonatal intraventricular hemorrhage. J. Neurosurg..

[19] Rosenberg GA, Estrada E, Kelley RO, Kornfeld M (1993). Bacterial collagenase disrupts extracellular matrix and opens blood-brain barrier in rat. Neurosci. Lett..

[20] Lekic T (2016). Cyclooxygenase-2 Inhibition Provides Lasting Protection Following Germinal Matrix Hemorrhage in Premature Infant Rats. Acta Neurochir. Suppl..

[21] Sabir H (2014). Combined treatment of xenon and hypothermia in newborn rats–additive or synergistic effect?. PLoS One.

[22] Sabir H, Cowan FM (2015). Prediction of outcome methods assessing short- and long-term outcome after therapeutic hypothermia. Semin. Fetal Neonatal Med..

[23] Nonato M, Gheler L, Balestrieri JV, Audi M, Prandini M (2019). Selective head cooling and whole body cooling as neuroprotective agents in severe perinatal asphyxia. Rev. Assoc. Med. Bras..

[24] Lekic T (2013). Evaluation of the hematoma consequences, neurobehavioral profiles, and histopathology in a rat model of pontine hemorrhage. J. Neurosurg..

[25] Zhang Y (2018). Biliverdin reductase-A attenuated GMH-induced inflammatory response in the spleen by inhibiting toll-like receptor-4 through eNOS/NO pathway. J. Neuroinflammation.

[26] Du XJ, Bathgate RA, Samuel CS, Dart AM, Summers RJ (2010). Cardiovascular effects of relaxin: from basic science to clinical therapy. Nat. Rev. Cardiol..

[27] Bathgate RA (2013). Relaxin family peptides and their receptors. Physiol. Rev..

[28] Teerlink JR (2017). Serelaxin in addition to standard therapy in acute heart failure: rationale and design of the RELAX-AHF-2 study. Eur. J. Heart Fail..

[29] Teerlink JR (2013). Serelaxin, recombinant human relaxin-2, for treatment of acute heart failure (RELAX-AHF): a randomised, placebo-controlled trial. Lancet.

[30] Teichman SL (2009). Relaxin, a pleiotropic vasodilator for the treatment of heart failure. Heart Fail. Rev..

[31] Maggioni AP (2019). Efficacy and safety of serelaxin when added to standard of care in patients with acute heart failure: results from a PROBE study, RELAX-AHF-EU. Eur. J. Heart Fail..

[32] Ghosh RK (2017). Serelaxin in acute heart failure: Most recent update on clinical and preclinical evidence. Cardiovasc. Ther..

[33] Dahlke M (2015). Safety and tolerability of serelaxin, a recombinant human relaxin-2 in development for the treatment of acute heart failure, in healthy Japanese volunteers and a comparison of pharmacokinetics and pharmacodynamics in healthy Japanese and Caucasian populations. J. Clin. Pharmacol..

[34] Moore XL (2007). Relaxin antagonizes hypertrophy and apoptosis in neonatal rat cardiomyocytes. Endocrinology.

[35] Nistri S (2012). Relaxin promotes growth and maturation of mouse neonatal cardiomyocytes *in vitro*: clues for cardiac regeneration. J. Cell Mol. Med..

[36] Feugang JM, Rodriguez-Munoz JC, Willard ST, Bathgate RA, Ryan PL (2011). Examination of relaxin and its receptors expression in pig gametes and embryos. Reprod. Biol. Endocrinol..

[37] Bagnell CA, Steinetz BG, Bartol FF (2009). Milk-borne relaxin and the lactocrine hypothesis for maternal programming of neonatal tissues. Ann. N. Y. Acad. Sci..

[38] Li P (2019). Rh-IFN-alpha attenuates neuroinflammation and improves neurological function by inhibiting NF-kappaB through JAK1-STAT1/TRAF3 pathway in an experimental GMH rat model. Brain Behav. Immun..

[39] Kobalava Z (2015). Pharmacokinetics of serelaxin in patients with hepatic impairment: a single-dose, open-label, parallel group study. Br. J. Clin. Pharmacol..

[40] Giam B (2018). Serelaxin attenuates renal inflammation and fibrosis in a mouse model of dilated cardiomyopathy. Exp. Physiol..

[41] Wang D (2017). Serelaxin improves cardiac and renal function in DOCA-salt hypertensive rats. Sci. Rep..

[42] Wilson BC, Connell B, Saleh TM (2006). Relaxin-induced reduction of infarct size in male rats receiving MCAO is dependent on nitric oxide synthesis and not estrogenic mechanisms. Neurosci. Lett..

[43] Bergeron LH (2015). Relaxin peptide hormones are protective during the early stages of ischemic stroke in male rats. Endocrinology.

[44] Szpecht D, Gadzinowski J, Seremak-Mrozikiewicz A, Kurzawinska G, Szymankiewicz M (2017). Role of endothelial nitric oxide synthase and endothelin-1 polymorphism genes with the pathogenesis of intraventricular hemorrhage in preterm infants. Sci. Rep..

[45] Leo CH, Jelinic M, Ng HH, Tare M, Parry LJ (2016). Time-dependent activation of prostacyclin and nitric oxide pathways during continuous i.v. infusion of serelaxin (recombinant human H2 relaxin). Br. J. Pharmacol..

[46] Stepien K, Tomaszewski M, Czuczwar SJ (2005). Neuroprotective properties of statins. Pharmacol. Rep..

[47] Dohare P (2016). AMPA-Kainate Receptor Inhibition Promotes Neurologic Recovery in Premature Rabbits with Intraventricular Hemorrhage. J. Neurosci..

[48] Dubner SE (2019). White matter microstructure and cognitive outcomes in relation to neonatal inflammation in 6-year-old children born preterm. Neuroimage Clin..

[49] Zarriello, S., Neal, E. G., Kaneko, Y. & Borlongan, C. V. T-Regulatory Cells Confer Increased Myelination and Stem Cell Activity after Stroke-Induced White Matter Injury. *J Clin Med* **8**, 10.3390/jcm8040537 (2019). 10.3390/jcm8040537 PMC6518209 31010132

[50] Kuban KCK (2019). Association of Circulating Proinflammatory and Anti-inflammatory Protein Biomarkers in Extremely Preterm Born Children with Subsequent Brain Magnetic Resonance Imaging Volumes and Cognitive Function at Age 10 Years. J. Pediatr..

[51] Young JM (2018). Altered white matter development in children born very preterm. Brain Struct. Funct..

[52] Klebe D (2017). Dabigatran ameliorates post-haemorrhagic hydrocephalus development after germinal matrix haemorrhage in neonatal rat pups. J. Cereb. Blood Flow. Metab..

[53] Chen W (2019). Modified behavioural tests to detect white matter injury- induced motor deficits after intracerebral haemorrhage in mice. Sci. Rep..

[54] Ng HH, Leo CH, Parry LJ (2016). Serelaxin (recombinant human relaxin-2) prevents high glucose-induced endothelial dysfunction by ameliorating prostacyclin production in the mouse aorta. Pharmacol. Res..

[55] Kilkenny C, Browne WJ, Cuthill IC, Emerson M, Altman DG (2010). Improving bioscience research reporting: the ARRIVE guidelines for reporting animal research. PLoS Biol..

[56] Manaenko A, Lekic T, Barnhart M, Hartman R, Zhang JH (2014). Inhibition of transforming growth factor-beta attenuates brain injury and neurological deficits in a rat model of germinal matrix hemorrhage. Stroke.

[57] Rolland WB (2013). Fingolimod reduces cerebral lymphocyte infiltration in experimental models of rodent intracerebral hemorrhage. Exp. Neurol..

[58] Fathali N, Lekic T, Zhang JH, Tang J (2010). Long-term evaluation of granulocyte-colony stimulating factor on hypoxic-ischemic brain damage in infant rats. Intensive Care Med..

[59] Zhou Y, Fathali N, Lekic T, Tang J, Zhang JH (2009). Glibenclamide improves neurological function in neonatal hypoxia-ischemia in rats. Brain Res..

[60] Plank AC (2018). Early Alterations in Operant Performance and Prominent Huntingtin Aggregation in a Congenic F344 Rat Line of the Classical CAGn51trunc Model of Huntington Disease. Front. Neurosci..

