# Supplementary material for: Serelaxin activates eNOS, suppresses inflammation, attenuates developmental delay and improves cognitive functions of neonatal rats after germinal matrix hemorrhage
Source: Sci Rep. 2020 May 15;10:8115. doi: 10.1038/s41598-020-65144-4 (PMC7229117; doi:10.1038/s41598-020-65144-4)

SUPPLEMENTAL MATERIAL

Serelaxin activates eNOS, suppresses inflammation, attenuates developmental delay and improves cognitive functions of neonatal rats after germinal matrix hemorrhage

Mingming Xu[1,4], Seyler L[2], Bäuerle T[2], Kalinichenko LS[3], Müller CP[3], Huttner HB[1], Schwab S[1], Manaenko A[1] ✉.

[1]Department of Neurology, University Clinic, Friedrich-Alexander-University Erlangen-Nuremberg, Erlangen, Germany.

[2] Department of Radiology, University Clinic, Friedrich-Alexander-University Erlangen-Nuremberg, Erlangen, Germany.

[3] Department of Psychiatry and Psychotherapy, University Clinic, Friedrich-Alexander-University Erlangen-Nuremberg, Germany.

[4] Department of Neurology, Xinhua hospital affiliated to Shanghai Jiaotong University School of Medicine, Shanghai, China.

✉ Corresponding author, electronic address: Anatol_Manaenko@outlook.com

Supplemental Fig. 1 Overall Study Design

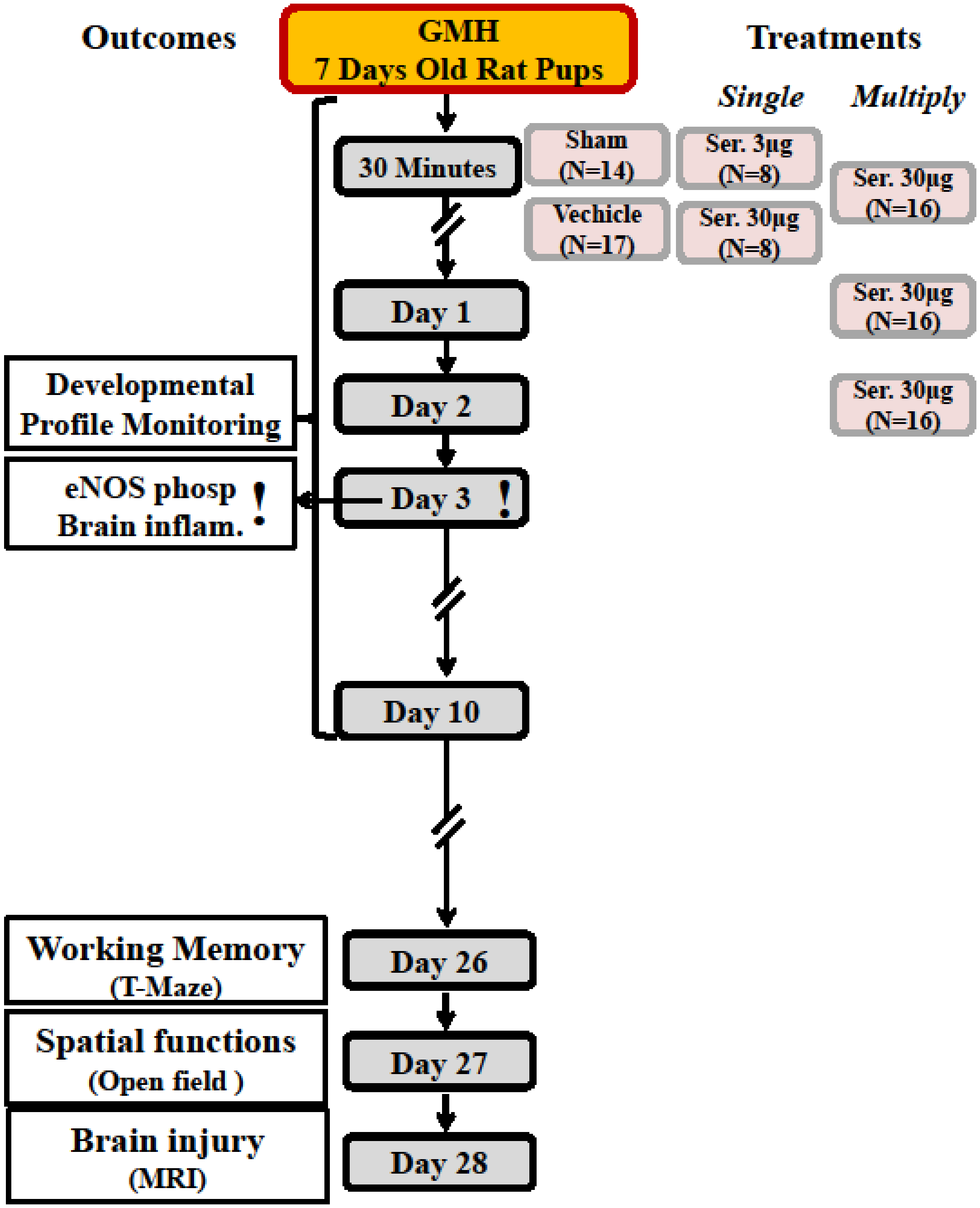

Supplemental Fig. 2

Representative MRI scans of animals` brains 28 days after GMH

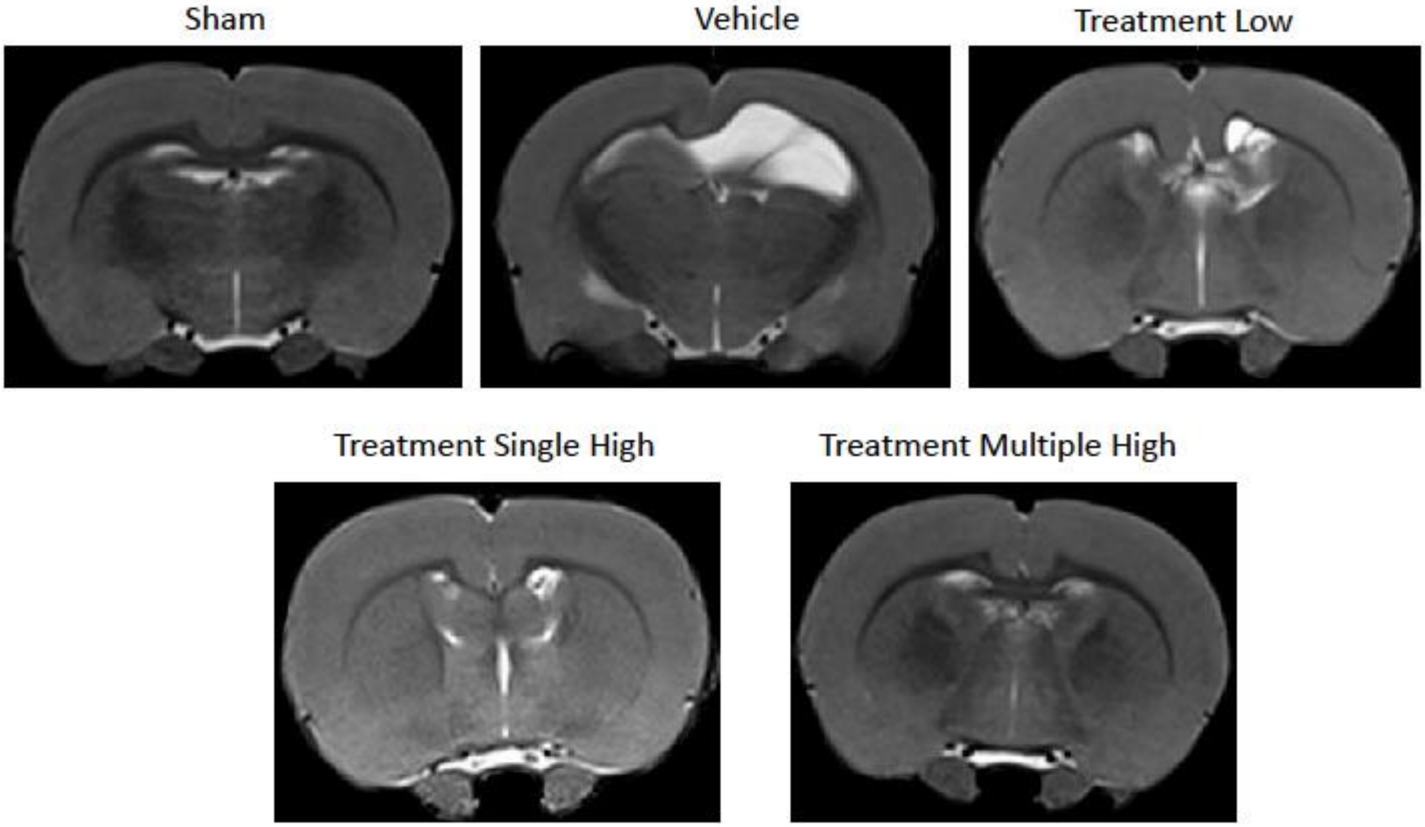

Supplemental Fig. 3

Results of western blot study.

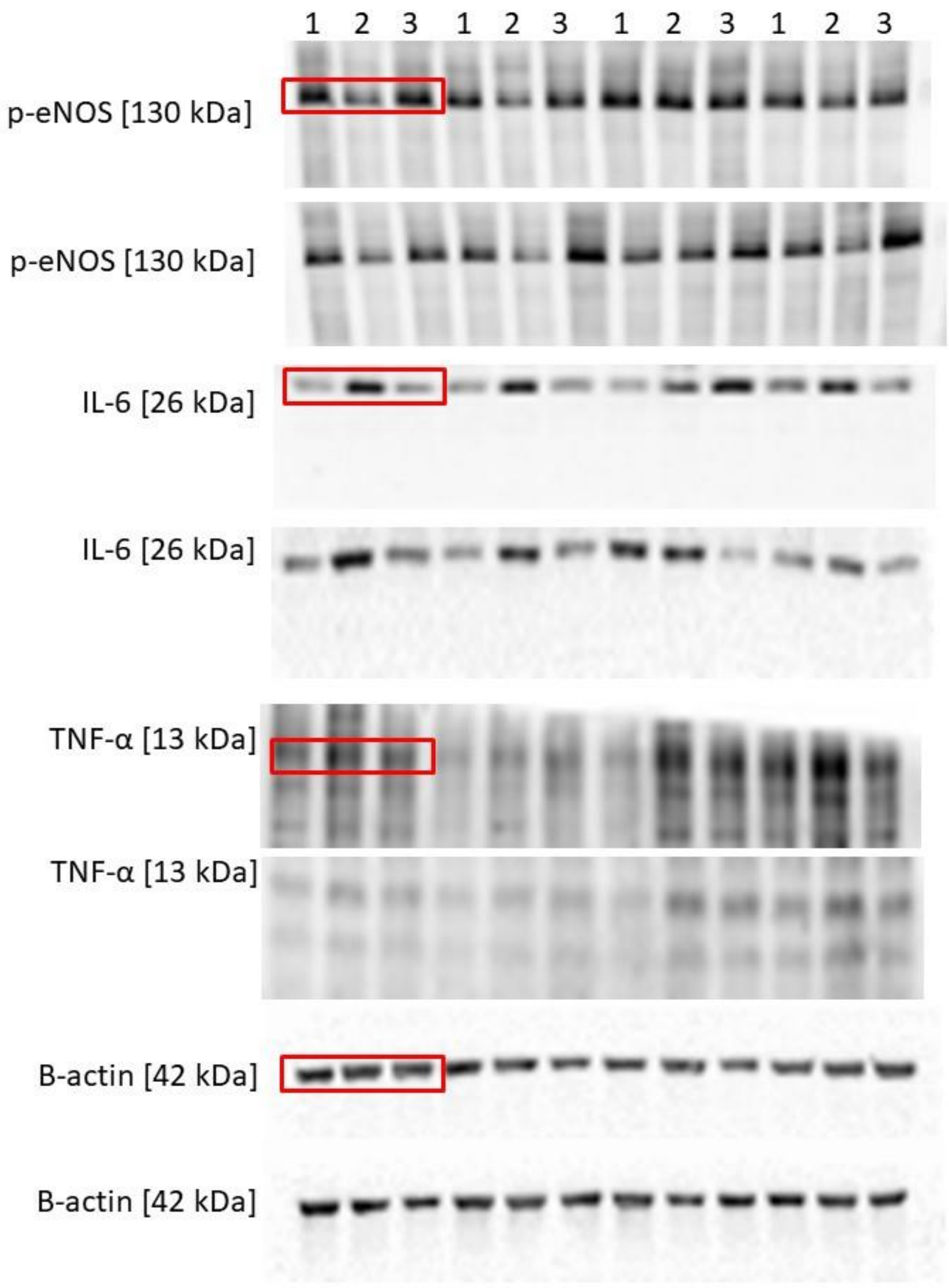

Supplement: Supplementary file 1 — Supplementary information. [file 41598_2020_65144_MOESM1_ESM.pdf]
